# New insights into Paeoniaceae used as medicinal plants in China

**DOI:** 10.1038/s41598-019-54863-y

**Published:** 2019-12-05

**Authors:** Xiaoxiao Zhang, Yuhui Zhai, Junhui Yuan, Yonghong Hu

**Affiliations:** Shanghai Key Laboratory of Plant Functional Genomics and Resources, Shanghai Chenshan Plant Science Research Centre, Chinese Academy of Sciences, Shanghai Institute of Jiangnan Tree Peony, Shanghai Chenshan Botanical Garden, Shanghai, 201602 China

**Keywords:** Biochemistry, Plant sciences

## Abstract

Paeoniaceae is an abundant germplasm resource with significant medicinal values in China, the principal medicinal components of which include paeoniflorin and paeonol. These compounds are typically obtained from air-dried root samples, which the use of freeze-drying as an alternative method has not been tested. Additionally, the presence of these two compounds in various wild Paeoniaceae species has not been previously explored, nor have the differences between various plant organs been fully evaluated. Here, freeze-drying and air-drying methods were compared to assess the changes in paeoniflorin and paeonol in root samples using ultra-performance liquid chromatography-mass spectrometer. The contents of these compounds in the roots, leaves, stems, and petals were then tested in freeze-dried materials. We also quantitatively detected the paeoniflorin and paeonol contents in the roots of 14 species collected from 20 natural habitats. Results indicated that the paeoniflorin content decreased under air-drying in comparison to freeze-drying, while the opposite trend was observed for paeonol. Our findings also demonstrated that the root xylem of species in Section *Moutan*, particularly *Paeonia ostii*, contains considerable paeonol and paeoniflorin and should thus be fully utilized as a medicinal resource. Furthermore, paeonol was mainly detected in the roots, while paeoniflorin was widely distributed in different organs; the highest content was in the leaf at the budding stage, suggesting that the leaves should be developed as a new paeoniflorin resource. Paeoniflorin contents were also found to be higher at earlier development stages. Based on the standards of the Chinese Pharmacopoeia, five species of Section *Moutan* and six species of Section *Paeonia* could be used as potential traditional Chinese medicinal resources. These findings of this study enhance our understanding of these two medicinal compounds and provide a foundation for the further development and utilization of Paeoniaceae as medicinal plant resources.

## Introduction

*Paeonia*, belonging to the monogeneric family Paeoniaceae, consists of 33 species of shrubs and perennial herbs mainly distributed in the temperate Eurasia, northwest Africa, and western North American^1^. In China, Paeoniaceae plants have been cultivated for more than 3,900 years. The shrubs, called tree peonies (Section *Moutan* DC.), have long been known as ‘King of flowers’, and the perennials, called herbaceous peonies (Section *Paeonia* Pan), were regarded as ‘Queen of Herbs’^2^. Paeoniaceae plants not only have high ornamental values but also exhibit significant medicinal applications. The use of tree peony and herbaceous peony as medicinal plants dates back to the medical book of the early Eastern Han Dynasty (25–220 A.D.). In the Pharmacopoeia of the People’s Republic of China, two traditional Chinese medicines, called “Mudanpi” and “Chishao”, are derived from the dry root phloem of tree peony and the dry roots of herbaceous peony, respectively^3^. Presently, *P*. *ostii* is widely cultivated in China for Mudanpi, while *P*. *lactiflora* is cultivated predominantly for Chishao. These two medicines both have the functions of clearing heat, cooling blood, promoting blood circulation, and removing blood stasis. While Mudanpi is largely used in the first two applications, Chishao is applied to the latter two. They also have been described to possess analgesic, sedative, anti-inflammatory, immunomodulatory, antioxidant, and antimicrobial properties, and they have been used as a remedy for cardiovascular and female genital diseases^4,5^. It has been confirmed that the principal medicinal ingredients of Mudanpi are paeoniflorin and paeonol, whereas that of Chishao is paeoniflorin^6,7^.

**Figure 1:**
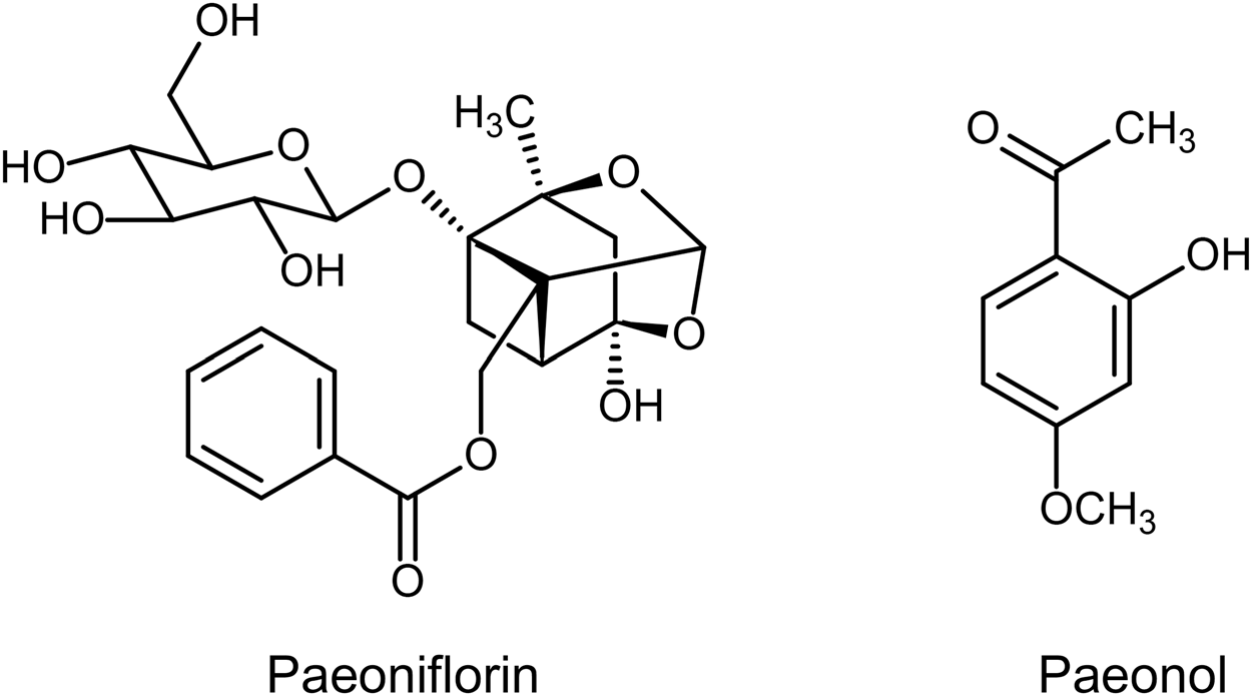
Chemical structure of paeoniflorin and paeonol.

Paeoniflorin is a monoterpene glucoside, and paeonol is a phenolic ketone (Fig. 1). These two compounds exhibit a variety of physiological and pharmacological activities^8,9^. The contents of bioactive compounds in plant tissues will vary during the air-drying process^10,11^, while immediate freeze-drying following collection best ensures that the compounds remain unchanged^12^. The root samples of Paeoniaceae plants have largely been air-dried in scientific researches^13,14^ and practical production^3^. However, studies concerning the changes in paeoniflorin and paeonol contents during air-drying are scarce. As secondary metabolites, paeoniflorin and paeonol are also influenced by various plant-related factors, such as age^15^, source organ^16^, and developmental stage^7^. Although some reports have assessed the impacts of these factors on paeoniflorin and paeonol contents, the plant samples in these studies were air-dried and thus might not accurately reflect the influence of these factors on paeoniflorin and paeonol contents in Paeoniaceae.

**Figure 2:**
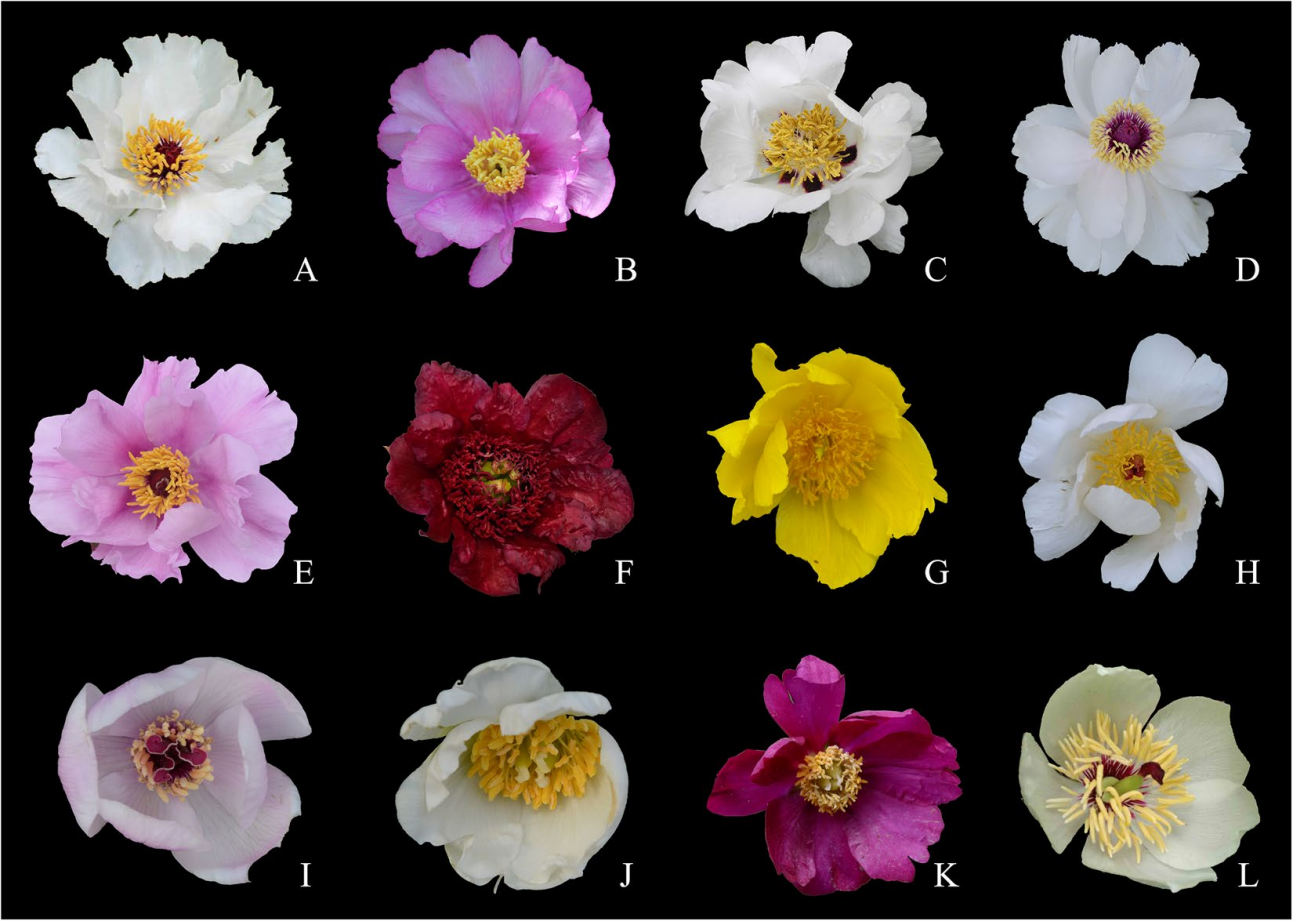
Some Paeonia species involved in the present study. (**A**) *P. jishanensis*; (**B**) *P. decomposita*; (**C**) *P. rockii*; (**D**) *P. ostii*; (**E**) *P. qiui*; (**F**) *P. delavayi*; (**G**) *P. ludlowii*; (**H**) *P. lactiflora*; (**I**) *P. mairei*; (**J**) *P. sterniana*; (**K**) *P. anomala*; (**L**) *P. obovata*.

The quantities of paeoniflorin and paeonol in Paeoniaceae are directly related to the medicinal efficacy^17,18^. The Pharmacopoeia thus clearly stipulates that the paeonol content should not be less than 1.2% in Mudanpi, and the paeoniflorin content in Chishao should not be less than 1.8%^3^. Paeonol is present in some Paeoniaceae plants, particularly in tree peonies^6,13,14^, while paeoniflorin occurs ubiquitously in all the species examined in this family^7,19,20^. There are abundant germplasm resources of Paeoniaceae in China^21^ (Fig. 2), and the roots of all Paeoniaceae plants are collected in their natural habitats for medicinal use by local pharmacists. It is believed that the roots have the same functions as Mudanpi or Chishao. Besides, in Tibet, Xinjiang, Sichuan, and Yunnan Provinces, local ethnic minorities also used the roots of native species to treat headaches, abnormal menstruation, hematuria, diarrhea, night sweats, and other diseases^22^. Previous studies have evaluated the medicinal ingredients of Paeoniaceae plants, but their root samples were obtained from plants that had been cultivated in the field for several years^14,23^ or from specimens that had been preserved for many years^13^. Moreover, the results from these studies were expressed as relative contents rather than absolute contents. In order to optimize Paeonia resource use, the root samples must be collected from the original habitats, and the results should be expressed as absolute contents.

In the present study, freeze-drying and air-drying methods were used to explore the changes in these two medicinal components in Paeoniaceae, which provided some suggestions for drying technology of Mudanpi and Chishao. The influence of plant age, organ, and developmental stage on paeoniflorin and paeonol contents were then investigated following freeze-drying. Furthermore, this study also evaluated the presence of these two principal medicinal ingredients in the roots of Paeoniaceae plants collected from natural habitats in China. Several new insights into these two bioactive compounds in Paeoniaceae are made, providing a theoretical basis for the development and utilization of Paeoniaceae plants as medicinal resources.

## Results and Discussion

### Effect of different drying methods on paeoniflorin and paeonol

In the *P*. *ostii* root phloem, *P*. *ostii* root xylem and whole root of *P*. *lactiflora*, the paeoniflorin contents under freeze-drying were higher than under the air-drying treatment (Table 1), decreasing by 81.25%, 46.40%, and 82.65%, respectively, under the latter. Paeoniflorin is unstable at high temperature^24^, which might explain the decrease in content during the air-drying process. Conversely, under the air-drying treatment, the paeonol contents in the phloem and xylem of *P*. *ostii* increased by 146.11% and 248.39%, respectively, compared with freeze-drying, which is contrary to the paeoniflorin results. The fresh root of tree peony contains paeonolide, and this compound is easily hydrolyzed into paeonol and L-arabinose under the action of enzymes^15^, which explains the higher paeonol content under air-drying than freeze-drying. The paeonol contents of *P*. *lactiflora* root under the two drying methods were both lower than 0.00 mg/g dw, and it indicated that paeonol content was really low in *P*. *lactiflora* root. These findings suggest that paeonol contents can be increased by using the air-drying method in the production of Mudanpi. Conversely, freeze-drying is better suited for increasing paeoniflorin contents in the production of Mudanpi and Chishao. The standard contents of paeoniflorin in Chishao and paeonol in Mudanpi in Pharmacopoeia were evaluated based on the air-drying method^3^. Thus, if the freeze-drying method is to be used, the standard contents of these two medicinal components in traditional Chinese medicine would need to be revised.

**Table 1:** Paeoniflorin and paeonol contents determined by different drying methods.

Sample	Compound	Freeze drying	Air drying	Rate of change (%)
*P*. *ostii* root phloem	Paeoniflorin (mg/g)	18.72 ± 1.24	3.51 ± 0.54	−81.27
	Paeonol (mg/g)	5.92 ± 0.34	14.57 ± 1.09	146.05
*P*. *ostii* root xylem	Paeoniflorin (mg/g)	21.27 ± 1.12	11.4 ± 0.81	−46.41
	Paeonol (mg/g)	1.86 ± 0.16	6.48 ± 0.27	247.9
*P*. *lactiflora* root	Paeoniflorin (mg/g)	44.6 ± 3.31	7.74 ± 0.32	−82.65
	Paeonol (mg/g)	0.00	0.00	/

In the production of Mudanpi, the xylem is usually discarded as agricultural waste. Our results indicated that although the paeonol contents in the xylem were lower than that of the phloem, the paeoniflorin contents in the xylem were higher than that in the phloem under both drying methods. *Paeonia ostii* is the most widely cultivated tree peony for medicinal use, and the annual output of Mudanpi is about 500 t, which accounts for half of the total output in China^15^. Therefore, a large amount of root xylem has been discarded during production, which is a great waste of this medicinal resource. In fact, the whole root of *P*. *ostii* could be directly used to produce Mudanpi. This would not only fully utilize the medicinal resource, but would also reduce the processing and economic costs of the production of Mudanpi.

### Effect of plant age on paeoniflorin and paeonol

Cultivated *P*. *ostii* of one to seven years of age and *P*. *lactiflora* of one to three years of age were used to investigate the effects of plant age on paeoniflorin and paeonol contents. The roots of *P*. *ostii* and *P*. *lactiflora* were each divided into phloem and xylem, and the paeoniflorin and paeonol contents were measured following freeze-drying.

**Figure 3:**
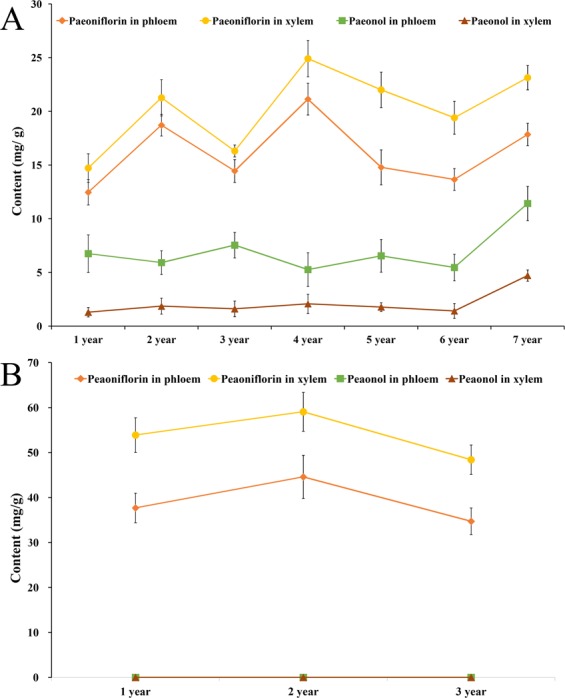
Variations in paeoniflorin and paeonol contents in one- to seven-year-old cultivated *P*. *ostii* (**A**) and one- to three-year-old *P*. *lactiflora* (**B**).

As far as *P*. *ostii* was concerned, the change trend of both paeoniflorin and paeonol contents had no visualization correlation with plant age (Fig. 3A). The paeoniflorin content in the xylem of four-year-old plants was the highest, reaching 24.92 mg/g dw, whereas the phloem of seven-year-old plants contained the highest paeonol content (11.42 mg/g dw). In contrast, a previous study^25^ demonstrated that the phloem of five-year-old plants had the highest paeonol content, which might be attributed to the different drying method used (air-drying). The paeoniflorin contents were always higher than the paeonol contents in both the phloem and xylem. On the contrary, Shi *et al*.^26^ and Li *et al*.^27^ both reported that the paeonol content was higher than the paeoniflorin content in the phloem of *P*. *ostii*, which might also be the result of the drying method used. Paeoniflorin and paeonol were conversely distributed in the roots of different-aged plants. Paeonol was present in higher quantities in the phloem, while paeoniflorin was more abundant in the xylem. These results imply that the root xylem of *P*. *ostii* should be fully utilized to take advantage of the high paeoniflorin content.

As observed in *P*. *ostii*, paeoniflorin was also mainly distributed in the xylem of *P*. *lactiflora* of three different ages and was higher than the paeonol content in both the phloem and xylem (Fig. 3B). The highest content of paeoniflorin was detected in the xylem of two-year-old plants, which was in accordance with the study of Sheng *et al*.^28^. In one- to three-year-old plants, the paeoniflorin contents in the xylem of *P*. *lactiflora* (48.42~59.07 mg/g dw) were significantly higher than those of *P*. *ostii* (14.73~24.92 mg/g dw). Similarly, the paeoniflorin contents in the phloem of *P*. *lactiflora* (34.72~44.60 mg/g dw) were also markedly higher than these of *P*. *ostii* (12.47~18.72 mg/g dw). This suggests that the roots of *P*. *lactiflora* may be the best paeoniflorin source. However, the paeonol contents in both the phloem and xylem of *P*. *lactiflora* were lower than 0.00 mg/g dw.

The correlation of paeoniflorin and paeonol in the xylem and phloem of one- to seven-year-old *P*. *ostii* plant roots was analyzed (Table 2). There was no significant correlation between paeoniflorin and paeonol content in both the phloem and xylem, and these findings were in accordance with a previous study^23^. The paeoniflorin content in the phloem was significantly correlated with that in the xylem at the *P* = 0.05 level, and a significant correlation was also observed between paeonol content in the phloem and xylem. These results demonstrated that the synthesis or transport of paeoniflorin and paeonol in both parts of the root were synchronized.

**Table 2:** Pearson correlation analysis between paeoniflorin and paeonol contents in different parts of *Paeonia ostii* roots in one- to seven-year-old.

	Paeoniflorin in phloem	Paeoniflorin in xylem	Paeonol in phloem
Paeoniflorin in xylem	0.834^*^		
Paeonol in phloem	0.013	0.071	
Paeonol in xylem	0.435	0.523	0.870^*^

^*^Correlation is significant at *P* = 0.05 level.

### Paeoniflorin and paeonol contents in different plant organs

The root is considered as the primary medicinal organ of Paeoniaceae plants, while the other tissues have been shown to contain a certain amount of medicinal ingredients^16,29,30^. In order to clarify the distribution of paeonol and paeoniflorin in different organs, the contents of these compounds in the leaf, petal, stem, root xylem, and phloem of *P*. *ostii* at the budding stage were determined after freeze-drying.

**Figure 4:**
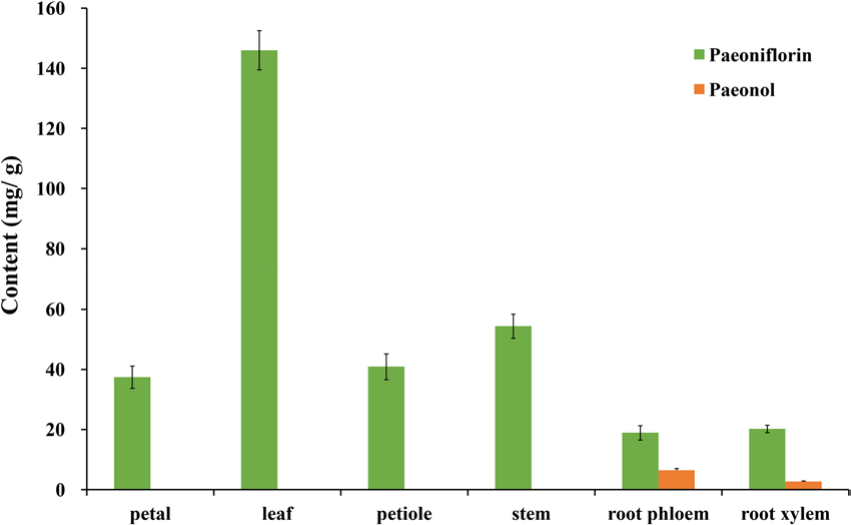
Variations in paeoniflorin and paeonol contents in different organs of cultivated *P*. *ostii*.

As shown in the Fig. 4, paeonol was mainly distributed in the roots, especially in the phloem. Paeonol was barely detected in the other organs, the contents were all lower than 0.00 mg/g dw. In contrast, the highest paeoniflorin content was found in the leaf (144.41 mg/g dw), whereas the lowest content was found in the root phloem (16.86 mg/g dw), i.e., the medicinal organ. During production, the farmers only harvest the root phloem of tree peony, while the other organs are discarded. Our results suggest that this is a great waste of plant resources. Furthermore, the extensive harvesting of plant roots could also damage the local ecological environment. Our study showed that the paeoniflorin content in the leaf was almost nine times higher than that of the root phloem at the budding stage. The leaves of tree peony at the fruit development stage^30^ and fruit maturity stage^31^ both contained considerable amounts of paeoniflorin, indicating that the leaf also has potential medicinal value. As a perennial woody shrub, tree peony is slow-growing, and its roots are used as medicine after four to five years of cultivation. However, the leaves can be harvested every year, and considerable biomass can be obtained. The development of the leaf as new resource of paeoniflorin can expand the industrial chain of tree peony and can also increase the economic income of farmers. Moreover, removing some leaves at appropriate stage can reduce the incidence of plant disease, which is also much more conducive to the healthy growth of tree peony.

### Effect of different developmental stages on paeoniflorin and paeonol

**Figure 5:**
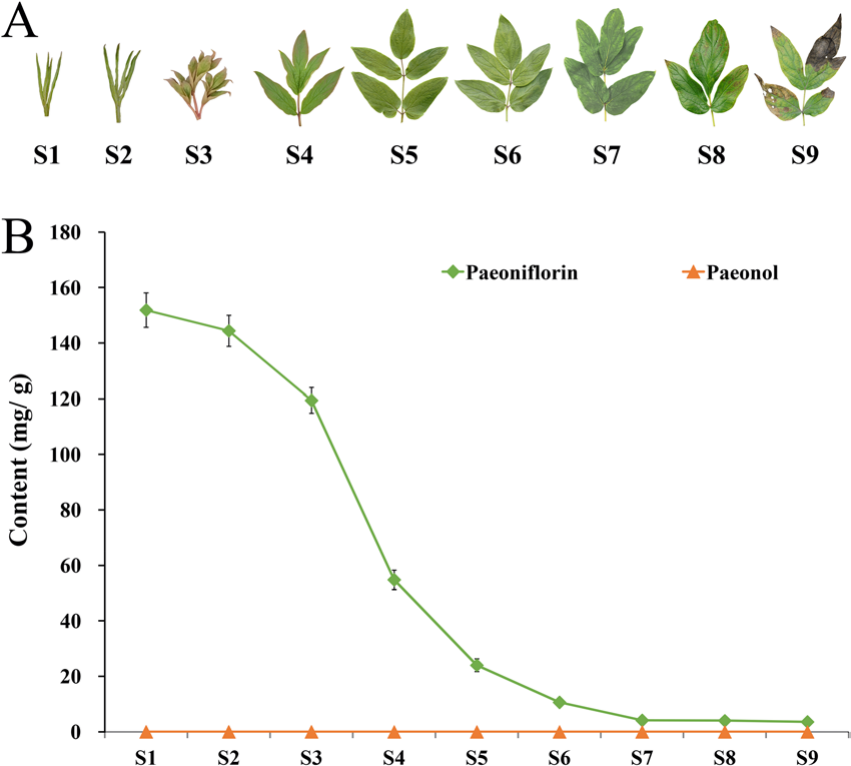
Variations in paeoniflorin and paeonol contents in the leaves of cultivated *P*. *ostii* at nine developmental stages. (**A**) Leaf shape characteristics of *P*. *ostii* at nine developmental stages (S1–S9); (**B**) Paeoniflorin and paeonol contents from S1 to S9.

Several studies have shown that the paeoniflorin and paeonol contents in the roots at different phenological stages are different^7,32^. However, these differences were not found to be significant, and no obvious trend was detected. This might be due to discrepancies in the sampling of root samples. In these studies, the root samples were collected continuously during a long period of time. In the case of collecting from the same plant, the growth of the individual could be affected, which would then impact root growth. If sampling from different plants, the paeoniflorin and paeonol contents could be affected by growth differences among the individuals. Furthermore, paeoniflorin and paeonol contents are also affected by the root circumference^33^. As a result of these factors, the leaf was selected as the experimental material to assess the changes in paeoniflorin and paeonol contents at different developmental stages (Fig. 5). The results can also provide a reference for the appropriate collection stage for the leaves.

Our findings showed that the paeoniflorin content was the highest at S1, reaching 151.87 mg/g dw (Fig. 5B). With plant growth, the paeoniflorin content decreased gradually until S9. At this stage, the paeoniflorin content was only 3.56 mg/g dw, approximately 2.3% of that at S1. Comprehensive analysis of the relationship between leaf status and paeoniflorin content showed that S1 to S4 was characterized by the process leaf curling to fully unfolding (Fig. 5A), during which the paeoniflorin content decreased rapidly, with the rate of decline increasing gradually. From S3 to S4 in particular, the decrement rate of paeoniflorin content was highest, which corresponded to the period of leaf expansion from half to full. From S4 to S9, the leaf matured gradually and then senesced. The decrement rate of paeoniflorin also gradually slowed, and the paeoniflorin contents at S7, S8, and S9 remained almost unchanged. These results suggested that the paeoniflorin content in the leaf was significantly correlated with developmental stage, with the paeoniflorin content being higher at earlier developmental stages. The paeoniflorin biosynthesis pathway has not been fully elucidated^34,35^. These differences in paeoniflorin content make the leaf a good system for a detailed investigation of the biosynthesis pathway. It has been reported that the leaves of Paeoniaceae plants are also rich in flavonoids^36^ and anthocyanins^37^. However, the leaf has not been effectively utilized in the production of medicinal preparations. To the best of our knowledge, this is the first report to determine the changes in the medicinal compounds at different developmental stages of tree peony leaves. A comprehensive assessment of the active ingredients and phenotypic characteristics suggests that the leaf, particularly at S3, should be developed as new health products or medicine. Unexpectedly, the paeonol contents of the nine stages were all lower than 0.00 mg/g dw, and thus no trends could be found.

### Paeoniflorin and paeonol contents in wild Paeoniaceae species

While numerous wild Paeoniaceae species exist in China, it remains unclear whether these species could be used in traditional Chinese medicines. In order to clarify the medicinal value of these wild resources, the paeoniflorin and paeonol contents were evaluated firstly. The roots of Section *Moutan* DC. species were also divided into the phloem and xylem for experimentation.

**Figure 6:**
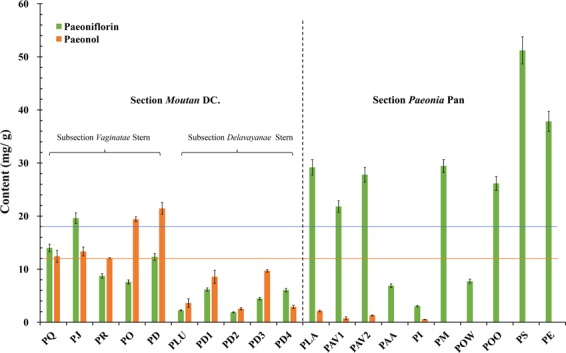
Variations in paeoniflorin and paeonol contents in the phloem of Section *Moutan* DC. species and the whole roots of Section *Paeonia* Pan species. See Table 3 for the population codes.

As shown in Fig. 6, the paeoniflorin and paeonol contents differed significantly among the 14 Paeoniaceae species. The root phloem of *P*. *decomposita* had the highest paeonol content (21.46 mg/g dw, 2.146%) of the nine species belonging to Section *Moutan* DC., while the lowest content (2.55 mg/g dw, 0.255%) was detected in the root phloem of *P*. *delavayi* (Chengjiang population). Han *et al*.^23^ reported that the paeonol contents of the root phloem from Section *Moutan* DC. ranged from 0.10% (*P*. *delavayi*) to 0.61% (*P*. *ostii*), while Guo *et al*.^13^ reported a range of 0.034% (*P*. *delavayi*) ~1.370% (*P*. *rockii*). These results differ from the results of the present study, which is probably due to differences in samples. The paeonol contents of the seeds from Section *Moutan* ranged from 1.49 mg/g dw (*P*. *lutea*) to 2.58 mg/g dw (*P*. *rockii*)^38^, indicating that paeonol is probably mainly distributed in the root. In the present study, the paeonol contents of five species belonging to Subsection *Vaginatae* were all higher than 1.2%, while those of four species belonging to Subsection *Delavayanae* were all lower than 1.2%. This indicated that the root phloem of *P*. *qiui*, *P*. *jishanensis*, *P*. *rockii*, *P*. *ostii*, and *P*. *decomposita* could be used as Mudanpi. However, harvesting of these roots could accelerate the extinction of these protected species^39,40^. The best approach for developing medicinal resources from these protected species involves the generation of new cultivars with high paeoniflorin and paeonol contents from these parent species.

There was significant variation in the paeoniflorin contents of the roots from seven species belonging to Section *Paeonia*, ranging from 3.06 mg/g dw (0.306%) for *P*. *intermedia* to 51.22 mg/g dw (5.122%) for *P*. *sterniana*. Guo *et al*.^13^ also detected the lowest content of paeoniflorin in the root of *P*. *intermedia* (1.030%), whereas the root of *P*. *mairei* (7.369%) had the highest content. He *et al*.^19^, Jin *et al*.^20^, and Yu and Xiao^41^ measured the paeoniflorin content in the roots of Section *Paeonia* plants native to China. The results were 0.00~10.72%, 1.11~7.78%, and 0.56~4.36%, respectively. However, as their classification is inconsistent with the current classification, it is not possible to directly compare the species. In the present study, the roots of *P*. *anomala* ssp. *anomala*, *P*. *intermedia*, *P*. *obovata* ssp. *willmottiae*, and *P*. *sterniana* contained less than 1.8% paeoniflorin, while the others species possessed more than 1.8% paeoniflorin. The highest paeoniflorin content was found in the root of *P*. *sterniana*, reaching 51.22 mg/g dw, which was almost three times that of the Pharmacopoeia standard. This suggests that the roots of certain wild Section *Paeonia* plants are more abundant in phytochemical resources than the cultivated plants. Furthermore, the roots of *P*. *lactiflora*, *P*. *anomala ssp*. *veitchii*, *P*. *mairei*, *P*. *obovata* ssp. *obovata*, *P*. *sterniana*, and *P*. *emodi* could be used as the traditional Chinese medicine Chishao. As suggested for the five species of Section *Moutan*, the best way of utilizing and protecting these species is via breeding to generate new cultivars.

Paeoniflorin was also found in the root phloem of Section *Moutan* species (Fig. 6). The paeoniflorin content in *P*. *jishanensis* reached 19.61 mg/g dw, which exceeded the 1.8% prescribed in the Pharmacopoeia. It is interesting that the contents of paeoniflorin in the root phloem of *P*. *qiui*, *P*. *jishanensis*, and *P*. *delavayi* (Yulong population) were even higher than that of paeonol. In the roots of seven Section *Paeonia* species, the paeonol contents were all at low levels. The maximum content was only 2.14 mg/g dw for *P*. *lactiflora*, while the content was even lower than 0.00 mg/g dw in *P*. *anomala* ssp. *anomala*, *P*. *mairei*, *P*. *obovata* ssp. *willmottiae*, *P*. *obovata* ssp. *obovata*, *P*. *sterniana*, and *P*. *emodi*. These results demonstrated that paeoniflorin is present in various Section *Moutan* and Section *Paeonia* species, while paeonol mainly exists in Section *Moutan* species^6,7,13,19,20^. Generally, the paeoniflorin contents in the roots of Section *Paeonia* species were higher than that of Section *Moutan* species, while the opposite trend was observed for paeonol contents. Moreover, the paeonol and paeoniflorin contents in the root phloem of Subsection *Vaginatae* species were higher than those of Subsection *Delavayanae*. These two medicinal ingredients appeared to be used as chemotaxonomic markers for Paeoniaceae species. Paeoniflorin was considered to occur naturally only in Paeoniaceae plants^41^. However, Choudhary *et al*.^42^ later isolated paeoniflorin from the water fern *Salvinia molesta*. In the present study, we did not detect paeoniflorin in *S*. *molesta* by UPLC-MS, nor did we detect it in another 16 plants from different families collected from the Shanghai Chenshan Botanical Garden (Supplementary Table S1). Our study thus confirms that paeoniflorin is the characteristic constituent of Paeoniaceae.

**Figure 7:**
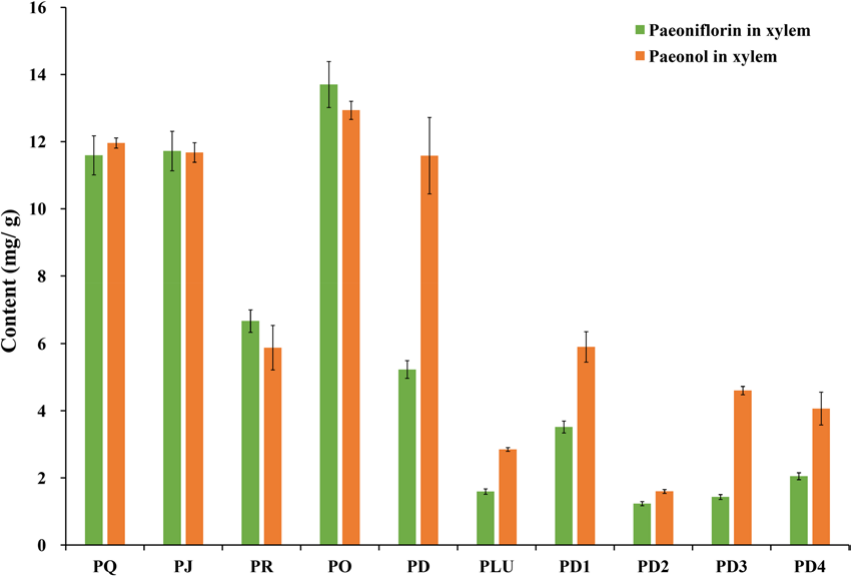
Variations in paeoniflorin and paeonol contents in the xylem of Section *Moutan* species. See Table 3 for the population codes.

Our study found considerable quantities of paeonol and paeoniflorin in the xylem of seven Section *Moutan* species (Fig. 7). The paeonol content in the xylem (12.94 mg/g dw) of *P*. *ostii* was higher than the standard in the Pharmacopoeia (1.2%), while those of *P*. *jishanensis* (11.68 mg/g dw), *P*. *qiui* (11.96 mg/g dw), and *P*. *decomposita* (11.58 mg/g dw) were around 1.2%, which is in line with the Pharmacopoeia standard. In addition, the paeoniflorin content in the xylem (13.70 mg/g dw) of *P*. *ostii* was 1.8 times higher than that in the phloem, which is consistent with that of the cultivated population. These results also showed that the root xylem, especially from *P*. *ostii*, should be fully utilized for medicinal purposes.

## Conclusion

Our results demonstrated that the type of drying method (freeze-drying versus air-drying) has a considerable impact on the paeoniflorin and paeonol contents in the roots of Paeoniaceae, with the former decreasing, and the latter increasing, under air-drying. It is therefore necessary that a suitable drying method is adopted in accordance with the production of traditional Chinese medicines, Mudanpi and Chishao. One significant finding was that the root xylem of Section *Moutan*, particularly from *P*. *ostii*, should be fully utilized for medicinal purposes instead of being discarded, as it contains considerable quantities of paeonol and paeoniflorin. Furthermore, the influences of three plant-related factors on the contents of paeonol and paeoniflorin under freeze-drying were evaluated for the first time in the present study. Firstly, we discovered that the contents of these two medicinal ingredients did not accumulate with age. Secondly, paeonol was mainly detected in the root, while paeoniflorin was widely distributed in various organs, with the highest content detected in the leaf at the budding stage. Thirdly, we found that the developmental stage was more earlier, paeoniflorin content was more higher. These findings suggested that the leaves of Paeoniaceae could be developed for medicinal use. As the paeoniflorin biosynthesis pathway has not been fully elucidated, our findings demonstrate that the leaf is a good system for a detailed investigation of the paeoniflorin biosynthesis pathway. Additionally, the absolute contents of paeonol and paeoniflorin in the roots of Paeoniaceae plants collected from natural habitats in China were also firstly reported. According to the standards stipulated in the Chinese Pharmacopoeia, the root phloem of five species belonging to Section *Moutan* and the roots of six species belonging to Section *Paeonia* could be used as the traditional Chinese medicines, Mudanpi and Chishao, respectively. We also preliminarily explored the application of paeonol and paeoniflorin as chemotaxonomic markers for Paeoniaceae species. However, a greater sample size is required for a more thorough assessment.

## Methods

### Chemicals and reagents

Paeoniflorin and paeonol were purchased from Sigma-Aldrich (St. Louis, MO, USA). HPLC-grade methanol and acetonitrile were obtained from Adamas Reagent, Ltd. (Shanghai, China). The water used in analysis was purified using a Milli-Q water purification system (Millipore, Billerica, MA, USA). All other chemicals used were of analytical grade and were obtained from Sinopharm Chemical Reagent Co. Ltd. (Shanghai, China).

### Plant materials

The cultivated *P*. *ostii* and *P*. *lactiflora* used in this study were cultivated at the Shanghai Chenshan Botanical Garden (121°10′14″E, 31°4′52″N). The root samples from 20 populations of 14 wild species were all collected directly from the source of their natural habitats in China (Table 3) during September to October in 2016, 2017, and 2018. The taxonomic classification was carried out by Professor Yonghong Hu, and all the voucher specimens were deposited in the Shanghai Key Laboratory of Plant Functional Genomics and Resources, China.

**Table 3:** Sampling locality information for the Paeoniaceae 14 species.

Population	Taxon	Sample locality	Longitude E (°) and Latitude N (°)	Elevation (m)
PQ	*Paeonia qiui* Y. L. Pei et D. Y. Hong	Xunyang County, Shaanxi	109.32/32.98	1558
PJ	*P*. *jishanensis* T. Hong et W. Z. Zhao Synonym: *P*. *spontanea* (Rehder) T. Hong et W. Z. Zhao	Yichuan Co., Shaanxi	110.38/35.83	1226
PR	*P*. *rockii* (S. G. Haw & L. A. Lauener) T. Hong et J. J. Li ex D. Y. Hong	Feng Co., Shaanxi	106.46/33.94	1386
PO	*P*. *ostii* T. Hong et J. X. Zhang	Lushi Co., Henan	111.11/34.02	828
PD	*P*. *decomposita* Hand.-Mass.	Maerkang Co., Sichuan	102.02/32.00	2504
PLU	*P*. *ludlowii* (Stern & Taylor) D. Y. Hong	Linzhi Co., Tibet	94.63/29.48	2958
PDE1	*P*. *delavayi* Franch. Synonym: *P*. *potaninii* Komarov	Yajiang Co., Sichuan	101.15/30.07	3127
PDE2	*P*. *delavayi* Franch. Synonym: *P*. *lutea* Delavay ex Franch.	Chengjiang Co., Yunnan	102.90/24.76	2760
PDE3	*P*. *delavayi* Franch. Synonym: *P*. *lutea* Delavay ex Franch.	Lanping Co., Yunnan	99.46/26.62	3004
PDE4	*P*. *delavayi* Franch.	Yulong Co., Yunnan	100.17/26.80	3015
PLA	*P*. *lactiflora* Pallas	Lveyang Co., Shaanx	106.21/33.22	1685
PAV1	*P*. *anomala* ssp. *veitchii* (Lynch) D. Y. Hong & K. Y. Pan	Feng Co., Shaanxi	106.36/34.10	1897
PAV2	*P*. *anomala* ssp. *veitchii* (Lynch) D. Y. Hong & K. Y. Pan	Wudu Co., Gansu	102.19/31.51	2771
PAA	*P*. *anomala* ssp. *anomala* Hong & Pan	Jimunai Co., Xinjiang	85.55/47.13	1955
PI	*P*. *intermedia* C. A. Meyer	Yumin Co., Xinjiang	82.72/45.75	2043
PM	*P*. *mairei* H. Léveillé	Xunyang Co., Shaanxi	109.17/33.00	1623
POW	*P*. *obovata* ssp. *willmottiae* (Stapf) D. Y. Hong & K. Y. Pan	Lveyang Co., Shaanx	106.11/33.25	1765
POO	*P*. *obovata* ssp. *obovata* D. Y. Hong & K. Y. Pan	Jiaohe Co., Jilin	127.44/43.57	462
PS	*P*. *sterniana* H. R. Fletcher	Bomi Co., Tibet	96.05/29.73	3103
PE	*P*. *emodi* Wallich ex Royle	Jilong Co., Tibet	85.33/28.31	2348

### Sample preparation

Mudanpi is obtained from the root phloem, whereas Chishao is from the whole root. Therefore, the roots of *P*. *ostii* was divided into the phloem and xylem. The fresh organs from cultivated *P*. *ostii* and *P*. *lactiflora*, including the petal, leaf, stem, and root, were immediately frozen in liquid nitrogen, and then freeze-dried using a vacuum freeze dryer (Modulyod-230, Thermo Electron Corporation, Waltham, USA). The roots of the wild-collected species were air-dried at 35°C for 72 h to achieve a constant weight. The dried organs were ground into a powder using an electrical grinder (JP-250A-8, Jiugong Economy and Trade Co. Ltd., Shanghai, China), and then sifted through a 380-μm mesh. The powders were placed in labeled plastic bags under vacuum and subsequently stored at −40 °C until extraction.

The extraction was performed according to the method developed by Zhang *et al*.^38^ with slight modifications. Approximately 1 g of the dried powder sample was homogenized and extracted with ultrasonic assistance in 20 mL of acidified methanol solution (1 M HCl in 80% methanol) for 30 min and maintained at 25 °C. The homogenate was centrifuged at 12,000 rpm for 10 min at 4 °C (Centrifuge 5810 R, Eppendorf, Germany). The supernatants were filtered through a 0.22-µm membrane filter before ultra-performance liquid chromatography-mass spectrometry (UPLC-MS) analysis.

### UPLC-MS analysis

Chromatographic analysis was performed on a Thermo Scientific Dionex UltiMate 3000 UPLC system equipped with diode array detector (DAD-3000), autosampler (WPS-3000TRS), column oven (TCC-3000RS), and pressure pump (HPG-3400RS). The column was a Thermo Scientific Syncronis C18 column (100 mm × 2.1 mm, 1.7 µm). The injection volume was 5 µL, and the column temperature was maintained at 45 °C. Samples were eluted at a flow rate of 0.35 mL/min under the following conditions (solvent A: water/formic acid 99.9:0.1, v/v; solvent B: acetonitrile): 0~1 min, 5% B; 1~18 min, 5~99% B; 18~22 min, 99% B; 22~23 min, 99~5% B; 23~25 min, 5% B. The wavelengths used for the quantification were 230 nm for paeoniflorin and 274 nm for paeonol. All of the samples were tested in triplicate and each of them was injected once.

Paeoniflorin and paeonol were identified using a Thermo Scientific Q Exactive Plus hybrid quadrupole-Orbitrap mass spectrometer equipped with a heated electrospray ionization (ESI) source. The dual ESI source operated in both positive and negative ionization modes, and the spray voltages were set to 3.5 KV and 2.5 KV, respectively. Sheath gas and aux gas were set to 40 units and 10 units, respectively. The S-lens RF level was 50. The capillary temperature was held at 320 °C and the aux gas heater temperature was adjusted to 350 °C. Full MS^1^ scans were acquired from a scan range of 100~1500 *m/z* in the Orbitrap at a resolution of 70,000, followed by the MS^2^ scans for the top 10 precursor ions at a resolution of 17,500 in a data-dependent acquisition mode and isolation window of 2.0 *m/z*. Data acquisition was performed with Xcalibur 4.0 software.

### Method validation

Paeoniflorin and paeonol were identified by comparing their retention times and MS spectra with those of the pure standards (Supplementary Figs. S1 and S2). Quantification was performed by using the external standard method. Eight-points calibration curves were used for each standard. The retention time, mass spectrum data calibration equation, linear ranges (mg mL^−1^), determination coefficients (*r*^2^), limit of detection and quantification (mg mL^−1^), and the intra-day and inter-day precisions for the analyses are shown in Supplementary Table S2. The results were expressed as milligrams per gram of dry weight (mg/g dw).

### Statistical analysis

All analyses were conducted in triplicate, and the results were expressed as the mean ± standard deviation (SD). One-way analysis of variance (ANOVA) and Duncan’s multiple range tests were used to analyze and determine the significance of the differences at a *P-*level of 0.05. A two-tailed Pearson’s correlation test was performed to determine the correlations between two ingredient contents and plant ages. All analyses were performed in Microsoft Excel and SPSS software (version 19.0 for Windows; SPSS Inc., 2010).

## Supplementary information

Supplementary information
