# Supplementary material for: New insights into Paeoniaceae used as medicinal plants in China
Source: Sci Rep. 2019 Dec 5;9:18469. doi: 10.1038/s41598-019-54863-y (PMC6895042; doi:10.1038/s41598-019-54863-y)
Supplement: Supplementary file 1 — Supplementary information [file 41598_2019_54863_MOESM1_ESM.pdf]

New insights into Paeoniaceae used as medicinal plants in China

Xiaoxiao Zhang, Yuhui Zhai, Junhui Yuan* & Yonghong Hu*

Shanghai Key Laboratory of Plant Functional Genomics and Resources, Shanghai Chenshan Plant Science Research Centre, Chinese Academy of Sciences, Shanghai Institute of Jiangnan Tree Peony, Shanghai Chenshan Botanical Garden, Shanghai, 201602, China

Correspondence and requests for materials should be addressed to J. Y. (email: yuanjunhuigsly@126.com) or Y. H. (email: huyonghong@csnbgsh.cn)

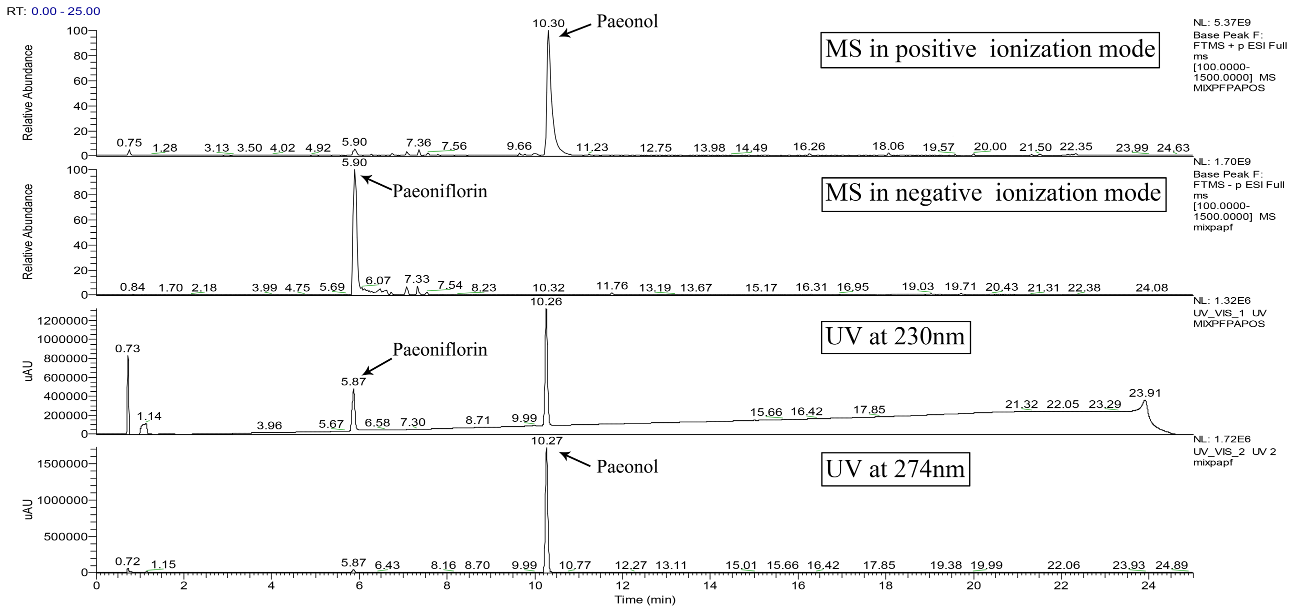

Supplementary Figure S1. Representative UPLC-MS trace of mixed references.

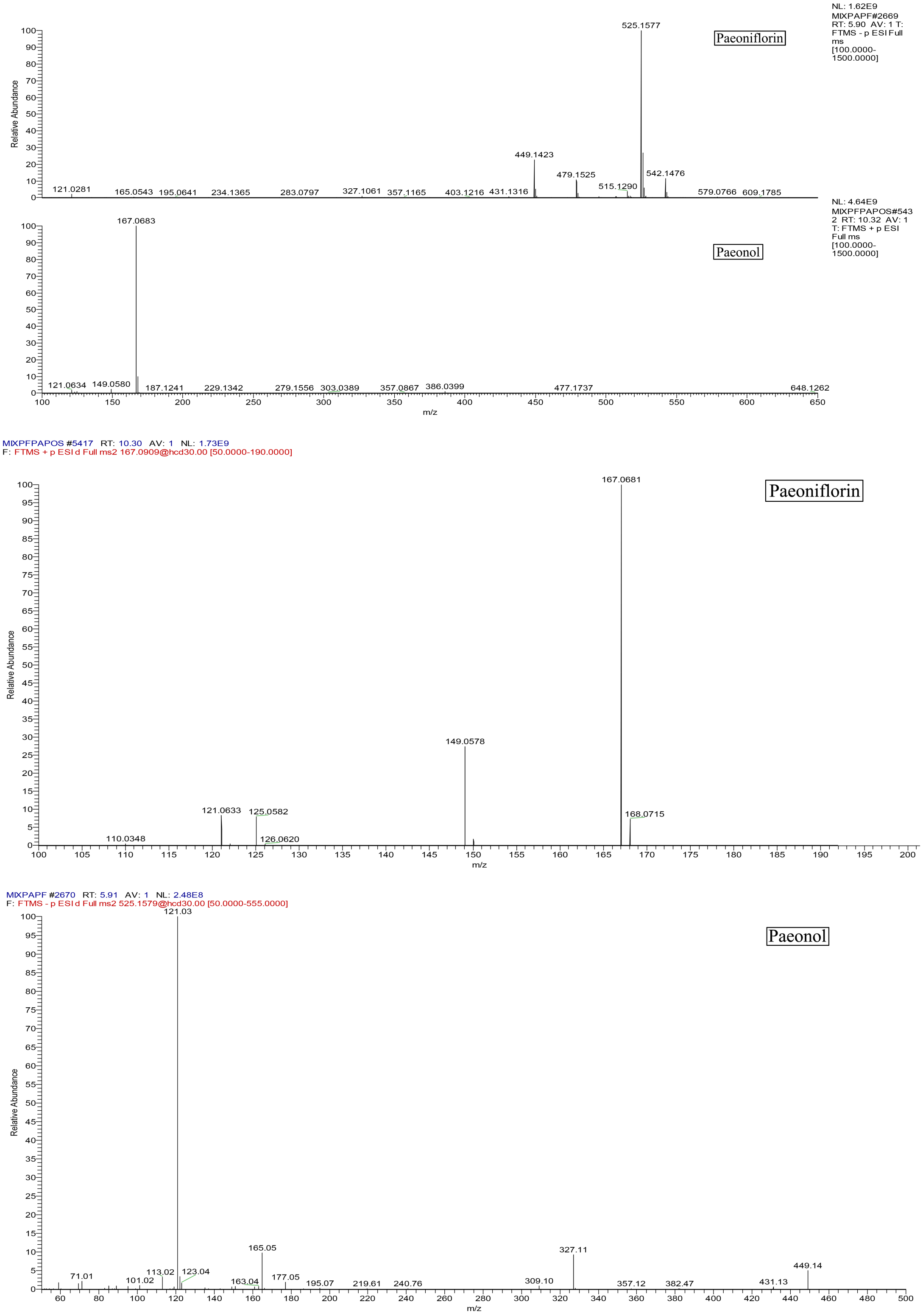

Supplementary Figure S2. MS spectra of paeoniflorin and paeonol.

Supplementary Table S1. The plant species in this study used for detection of paeoniflorin.

No.	Species	Family	Organ
1	*Salvinia molesta*	Saliviniaceae	Leaf, root
2	*Ilex cornuta*	Aquifoliaceae	Leaf
3	*Ilex chinensis*	Aquifoliaceae	Leaf
4	*Pyracantha fortuneana*	Rosaceae	Leaf
5	*Photinia serrulata*	Rosaceae	Leaf
6	*Euonymus myrianthus*	Celastraceae	Leaf
7	*Cyclobalanopsis myrsinifolia*	Fagaceae	Leaf
8	*Ligustrum japonicum*	Oleaceae	Leaf
9	*Acer pseudosieboldianum*	Aceraceae	Leaf
10	*Cephalotaxus sinensis*	Cephalotaxaceae	Leaf
11	*Narcissus tazetta*	Amaryllidaceae	Leaf
12	*Camellia japonica*	Theaceae	Leaf
13	*Magnolia delavayi*	Magnoliaceae	Leaf
14	*Mahonia fortunei*	Berberidaceae	Leaf
15	*Hydrocotyle sibthorpioides*	Apiaceae	Leaf
16	*Epipremnum aureum*	Araceae	Leaf

Supplementary Table S2. Experimental data of paeoniflorin and paeonol.

	Paeoniflorin	Paeonol
t_R/min	5.87	10.27
$[M+HCOO]^-$ *m/z*	525.1577	/
$[M+H]^+$ *m/z*	/	167.0683
Calibration equation[a]	y = 12425.5259 x + 938.0193	y = 45191.3413 x + 313.5921
r^2	0.9990	0.9994
Linear range (mg mL^{-1})	0.06-5.58	0.01-1.21
LOD[b] (mg mL^{-1})	0.12	0.22
LOQ[c] (mg mL^{-1})	0.24	0.34
Intra-day RSD[d] (%) (n = 6)	2.38	1.46
Inter-day RSD (%) (n = 6)	2.12	1.69
Recovery and RSD (%) (mean, n = 6)	101.61, 2.21	98.86, 1.31

[a] y, peak area; x, concentration of compound (mg mL^{-1}); [b] LOD=limit of detection, S/N=3; [c] LOQ=limit of quantification, S/N=10; [d] RSD (%) = (standard deviation/ mean) ×100.
